# Risk factors for interruption in treatment among HIV-infected adolescence attending health care and treatment clinics in Tanzania

**DOI:** 10.1186/s12981-023-00512-4

**Published:** 2023-03-30

**Authors:** Expeditho L. Mtisi, Stella E. Mushy, Simon G. Mkawe, Antony Ndjovu, Eric Mboggo, Boniface S. Mlay, Frida Ngalesoni, Aisa Muya

**Affiliations:** 1 Amref Health Africa, Ali Hassan Mwinyi Road Plot 1019 P O, Box 2773, Dar es Salaam, Tanzania; 2 Department of General Studies, Dar es Salaam Institute of Technology, P O Box 2958, Dar es Salaam, Tanzania; 3 Department of Community Health Nursing, School of Nursing, Muhimbili University of Health and Allied Sciences, P O Box 65454, Dar es Salaam, Tanzania; 4 Department of Development Studies, School of Public Health and Social Sciences, Muhimbili University of Health and Allied Sciences, P O Box 65454, Dar es Salaam, Tanzania; 5 Department of Care and Treatment, National AIDS Control Program (NACP), Ministry of Health, P.O Box 784, Dodoma, Tanzania

**Keywords:** Risk factors, Interruption in treatment (IIT), HIV-infected, Antiretroviral therapy (ART), Adolescence, Care and treatment

## Abstract

**Background:**

Interruption in Treatment (IIT) is a challenge in HIV care and treatment programs in sub- Saharan Africa. High IIT among HIV adolescents has both individual and potential public health consequences including discontinuation of treatment, increased HIV transmission and risk of death. In this era of test and treat policy it is important to ensure that patients remain connected to HIV clinics to enable achieve UNAIDS 95-95-95 targets timely. This study aimed to assess risk factors for IIT among HIV-positive adolescence in Tanzania.

**Methods:**

We conducted retrospective longitudinal cohort study using secondary data of adolescent patients enrolled in care and treatment clinics in Tanga from October 2018 to December 2020. We defined Interuption in Treatment as missing clinic visits for 90 consecutive days after the last scheduled appointment date on anti-retroviral therapy (ART). Cox proportional hazard regression models were employed to identify risk factors of the outcome variable.

**Results:**

Among 2,084 adolescents of age between 15 and 19 years were followed for two years, whereby 546 (26.2%) had interrupted treatment. The median age of the participants was 14.6 years (interquartile range, IQR: 12.6–16.6 years), with age between 15 and 19 years, male sex, with advanced HIV disease and were not on Dolutegravir (DTG) related regimens were associated with interruption in treatment; (Hazard ratio (HR) 1.43, 95% CI: 1.23–1.66, p < 0.0001, HR 2.47, 95% CI: 1.62–3.77, p < 0.0001, HR: 2.47, 95% CI: 1.91– 3.21, p < 0.0001 and HR: 6.67, 95% CI: 3.36– 7.04, p < 0.0001 respectively). Adolescents who were on ART for less or equal one year compared to those on ART for more than one year were protective toward interruption in treatment (HR: 0.68, 95% CI: 0.54–0.87, p = 0.002).

**Conclusions:**

The risk of interruption in treatment was high among adolescents in HIV care and treatment facilities in Tanga. This might lead to poor clinical outcomes, and increased drug resistance among ART-initiated adolescents. Placing more adolescents with DTG based drug, strengthening access to care and treatment and rapid tracking of patients is recommended to improve patient outcomes.

## Introduction

Adolescents are among the populations most impacted by the global Human Immunodeficiency Virus (HIV) epidemic. Accoding to UNAIDS report an estimate of 1.6 million adolescents aged 10 to 19 were living with HIV worldwide by 2018 [1].

While the rollout of antiretroviral therapy (ART), and now including immediate access to all who test positive (test and treat), has brought much excitement and hope to both patients and practitioners, it has also brought many new questions and challenges, particularly regarding interruption in treatment(IIT). Interruption in treatment commonly known as Loss to follow-up among patients with HIV infection has both individual and potential public health consequences including discontinuation of treatment, increased HIV transmission and risk of death [2–4].

Studies show that interruption in treatment rate in Sub-Saharan African countries among adolescents ranged between 15% and 54% [5]. For instance, a study in Nigeria reported the probabilities of loss to follow up among adolescents living with HIV (ADLHIV) were 3.6%,6.9,% and 35.9% at 6,12, and 25 months respectively [6]. A similar study conducted in Kenya showed youth from 15 to 21 years of age over half (57%) had interrupted treatment of whom 26% interrupted treatment were observed immediately after enrollment [6]. Factors associated with increased risk of interruption in treatment among ADLHIV reported in various studies included; ADLHIV aged 15–19 years, male adolescents, those with HIV/TB co-infection history, those with malnutrition, having advanced WHO clinical stage, among adolescents who had prior exposure to ART and those who attended clinics at public health facilities [7–9].

In Tanzania, various studies and routine assessments of HIV services have been done among adults but limited studies exist among adolescents group [10]. A recent study conducted by Tesha et al., 2022 using data from National AIDS Control program (NACP) reported 42% rate of interruption in treatment among adolescents aging between 10 and 19 years in Tanzania.

Routine HIV care and treatment implementation program indicates a high rate of loss to follow-up among adolescents in various regions of Tanzania [11]. To achieve the 2030 goal of ending the HIV epidemic as a public health threat, identification of predictors of interruption in treatment is urgently needed to inform effective strategies of retention in care among adolescents living with HIV/AIDS since high rates of loss to follow-up diminish treatment options and substantially limit the effectiveness of ART strategies [12]. This study aimed at identifying risk factors for interruption in treatment among HIV-infected adolescents attending health care and treatment clinics in Tanga region, Tanzania.

## Methods

The current study used secondary data analysis from the study that was conducted from October 2018 to December 2020 among serologically confirmed HIV-infected adolescent patients. Inclussion criteria involved adolescents aged 10–19 years on antiretroviral therapy (ART) in the selected care and treatment districts health services facilities in Tanga supported by Amref Health Africa in Tanzania (Amref Tanzania), and who had at least one clinic visit after enrolment or ART initiation.

From the CTC2 database, we first extracted all HIV positive patients on ART, then obtained adolescents aged 10–19 years. A total of 2104 adolescent patients on ART from 86 districts in Tanga region were obtained. Twenty participants whose follow up visits data was not available were excluded from the study. After data cleaning, a total of 2084 adolescents were included in the final analysis.

The primary outcome of interest was interruption in treatment (IIT). IIT among patients on ART defined as missing clinic visits for 90 consecutive days or more after the last scheduled appointment date. Data from patients known to have died or been transferred out to a clinic not affiliated with Amref Tanzania were considered censored. These definitions are consistent with the 2011 World Health Organization (WHO) working group definitions [13].

The IIT variable was a binary variable categorized as clients who interrupted treatment by not appearing consecutively to their next scheduled appointments for more than 90 days and those who did not interrupt treatment. We considered the following variables as potential predictors of interruption in treatment: ART duration ( < = 1, > 1years); sex (male, female); age (10 to 14, 15 to 19 years); married (Cohabiting, Divorced, Married, single and Widow); WHO HIV disease stage (I, II, III, IV); BMI (< 18.5, 18.5 -<25, 25 - <30, 30+); pregnant (no/yes); district of residence (Handeni, Kilindi, Korogwe, Lushoto, Mkinga, Muheza, Pangani and Tanga); ART regimen (first line, second line); Viral load supreesion(suppressed, non supressed); DTG based drug (no/yes). The cut points for some covariates were determined based on the distribution of data to ensure that each category had enough data.

Descriptive statistical analyses were carried out to report demographic and clinical characteristics and baseline and last clinic visit for the whole study population. Categorical variables are described by number and percent for each category whereas continuous variables are described by medians and interquartile ranges (IQR).

Cox proportional hazard regression models were employed to determine hazard ratio of patients interruption in treatment with associated 95% confidence interval (CI). Kaplan-Meier (KM) curves were used to estimate the cumulative incidence of loss to follow-up from the date of ART initiation. Follow-up time ended at the minimum of the dates of death and last clinic visit. Patients were considered censored if they died or transferred out, or if the time from their last visit to the administrative cutoff of the study was less than that required by the definition of loss to follow-up. Potential risk factors that were statistically significant at a p-value of 0.2 or less in univariate analysis were included as potential confounders in multivariate models. All significance tests were two-sided, and differences were considered significant at p-value less than or equal 0.05. The wald p-value is a test for trend. The missing indicator method was used to handle missing data. Statistical analysis was performed using the SAS ® statistical software package, Release 9.3 (Cary, North Carolina, USA).

## Results

A total of 2104 adolescent patients were enrolled in a study and all were receiving ART during the study peiod. Of them, 20 (0.9%) were excluded because they never came back after enrollment leaving 2084 adolescents who attended clinic at least once after enrolment. A total of 546 adolescents (26.2%) had interrupted treatment during the two years study period.

The median age of the participants was 14.6 (interquartile range, IQR: 12.6–16.6 years) and 53% of the study population were female.

**Table 1:** Basic characteristics of the study population (N = 2,084)

Characteristic	N (%)
Age group (yrs)	
Median (IQR) 10–14 15 – 19	14.6 (12.6–16.6) 1073 (51.5) 1011 (48.5)
Sex	
Female Male	1106 (53.0) 978 (47.0)
District	
Handeni Kilindi Korogwe Lushoto Mkinga Muheza Pangani Tanga	309 (14.8) 95 (4.6) 457 (21.9) 309 (14.8) 153 (7.3) 223 (10.7) 104 (5.0) 434 (20.8)
BMI, kg/m^2^	
<18.5 18.5 – <25 25 – <30 30+	257 (12.3) 1775 (85.2) 10 (0.5) 42 (2.0)
WHO Stage	
I II III IV	317 (18.8) 249 (14.8) 926 (55.0) 192 (11.4)
Pregnant Status	
No Yes	903 (97.7) 21 (2.3)
Marita Status	
Cohabiting Divorced Married Single Widow	331 (17.3) 4 (0.2) 63 (3.3) 1502 (78.7) 9 (0.5)
ART duration (yrs)	
<=1 >1	343 (16.6) 1741 (83.4)
Adherence	
Good Poor	1936 (97.0) 60 (3.0)
VLR suppression	
Suppressed Not suppressed	1904 (91.4) 180 (8.6)
TB history	
No Yes	2073 (99.5) 11 (0.5)
DTG related drug	
No Yes	477 (23.0) 1607 (77.0)
Drug Type	
First line Second line	1994 (98.0) 39 (2.0)
Interruption in treatment	
No Yes	1538 (73.8) 546 (26.2)

Majority of the adolescence came from Korogwe and Tanga districts about 22% and 21% respectively, where Pangani region had only 5% of the participants. At the time of study, more than 61% of the participants were in WHO stage 3 or 4, and 85.2% had a normal BMI (18.5 - <25) where 91.4% had a successful viral load suppression. Among the study partcipants 1741 (83.4%) were initiated on ART for more than one year from the time of enrolment. At the time of study, 77% of the participants had placed in DTG based drug combination. Among the adolescents, 11(0.5%) had a history of tuberculosis infection during their life time. 17% (17%) of the adolescents were cohabiting, and 3.3% were married while 2.3% were pregnant at the time of study. Of the participants 180 (8.6%) presented with viral load suppression failure, and 98% of the participants were in first line regimen. Additionally 60 (3%) participants were poorly adhering to care and treatment.

**Fig. 1:**
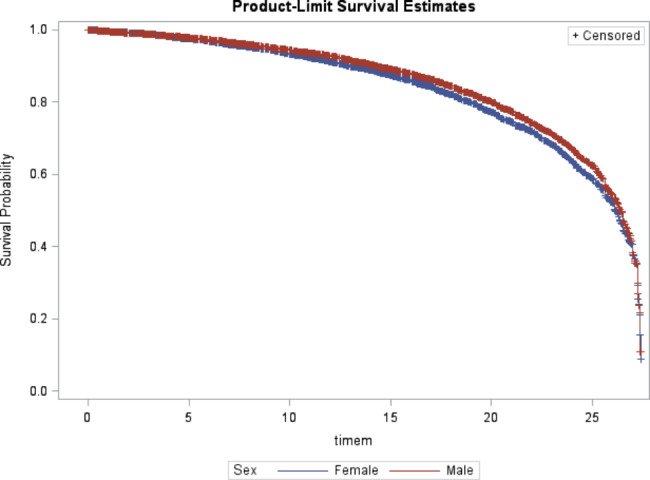
Survival probability among adolescents by Sex

**Fig. 2:**
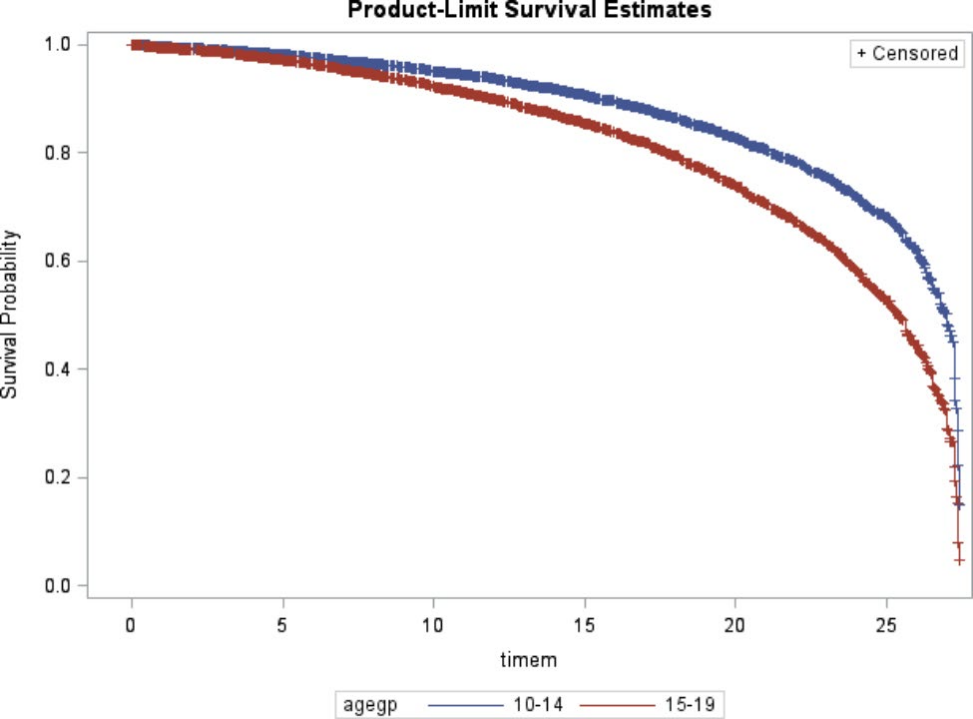
Survival probability among adolescents by age group

Covariate-adjusted Kaplan Meir plots for the overall interruption in treatment by sex and age group suppression categories, are presented in Fig. 1, and 2.

Table 2 provides a summary of risk factors associated with interruption in treatment among study population. In the multivariate model, adolescents aging 15–19 years had significantly increased risk of interruption in treatment (HR: 1.43, 95% CI: 1.23–1.66, p < 0.0001) compared to younger adolescents between 10 and 14 years of age. Male adolescents experienced more risk of interruption in treatment compared to female patients (HR: 2.47, 95% CI: 1.62–3.77, p < 0.0001) as well adolescents residing from Mheza district showed likelihood of interruption in treatment with (HR: 2.01, 95% CI: 1.52–2.65, p = 0.03) compared to those from Tanga district. Adolescents with WHO HIV stage IV had shown an independent significantly increased risk of interruption in treatment (HR: 2.47, 95% CI: 1.91–3.21, p = 0.003) compared to those with HIV stage I. There was no independent association of interruption in treatment among adolescent patients with low BMI (< 18.5 kg/m^2^) compared to those with normal BMI (18.5 -<25 kg/m^2^) (HR: 0.85, 95% CI: 0.71–1.04, p = 0.20).

Patients who were initiated ART within one year were more likely to be significantly protective toward interruption in treatment compared to patients who were on ART for more than one year (HR: 0.68, 95% CI: 0.54–0.87, p = 0.002). It was found that married adolescents had an increased risk of interruption in treatment compared to single with (HR: 1.59, 95% CI: 0.99–2.56, p = 0.35), however these results were not statistically significant. There was no association of interruption in treatment among those with poor adherence to treatment and adolescents who have ever had TB history (HR: 1.12, 95% CI: 0.75–1.66, p = 0.58), (HR: 1.72, 95% CI: 0.58–4.67, p = 0.32) respectively.

**Table 2:** Risk factors for interruption in treatment among Adolescence patients on ART in Tanga region

Characteristics	Univariate HR 95% CI	P for trend	Multivariate HR 95% CI	P for trend
Age group (yrs)		< 0.0001		< 0.0001
10 – 14 15 – 19	Reference 1.88 (1.77–1.99)		Reference 1.43 (1.23–1.66)	
Sex		< 0.0001		< 0.0001
Female Male	Reference 0.87 (0.82–0.92)		Reference 2.47 (1.62–3.77)	
District		0.002		0.03
Handeni Kilindi Korogwe Lushoto Mkinga Muheza Pangani Tanga	1.13 (1.02–1.26) 0.70 (0.57–0.85) 1.31 (1.19–1.44) 0.99 (0.89–1.11) 0.73 (0.62–0.86) 6.70 (6.06–7.41) 0.69 (0.57–0.88) Reference		1.18 (0.93–1.50) 1.33 (0.92–1.91) 0.55 (0.44–0.69) 0.35 (0.26–0.45) 0.63 (0.45–0.90) 2.01 (1.52–2.65) 0.79 (0.52–1.19) Reference	
BMI group, kg/m^2^		0.004		0.20
<18.5 18.5 – <25 25-<30 30+	0.66 (0.62–0.72) Reference 1.34 (0.97–1.87) 1.38 (1.16–1.65)		0.85 (0.71–1.04) Reference 3.80 (2.00–5.20) 0.62 (0.28–1.35)	
WHO Stage		< 0.0001		0.003
I II III IV	Reference 0.79 (0.70–0.90) 1.50 (1.36–1.64) 2.17 (1.93–2.45)		Reference 0.50 (0.38–0.66) 0.86 (0.70–1.05) 2.47 (1.91–3.21)	
Pregnant Status		0.70		
No Yes	Reference 1.09 (0.71–1.68)			
Marita Status		0.03		0.35
Cohabiting Divorced Married Single Widow	0.50 (0.45–0.56) 3.03 (1.60–5.75) 1.64 (1.37–1.95) Reference 1.70 (1.20–2.47)		0.82 (0.65–1.03) 5.03 (1.60–5.75) 1.59 (0.99–2.56) Reference 0.50 (0.20–1.28)	
ART duration (yrs)		< 0.0001		0.002
<=1 >1	2.27 (2.10–2.45) Reference		0.68 (0.54–0.87) Reference	
Adherence		< 0.0001		0.58
Good Poor	Reference 1.51 (1.31–1.73)		Reference 1.12 (0.75–1.66)	
TB history		0.0007		0.32
No Yes	Reference 1.85 (1.30–2.63)		Reference 1.72 (0.58–4.67)	
Regimen		< 0.0001		0.001
First line Second line	Reference 4.89 (4.10–5.85)		Reference 0.50 (0.34–0.76)	
Viral load suppression		< 0.0001		0.73
Suppressed Not suppressed	Reference 1.58 (1.45–1.73)		Reference 1.04 (0.83–1.30)	
Dolutegravir (DTG) related drug		< 0.0001		< 0.0001
No Yes	5.38 (4.24–6.53) Reference		6.67 (3.36–7.04) Reference	

Adolescents patients placed in second line regimen were significantly independently less likely to become interrupted in treatment (HR: 0.50, 95% CI 0.34–0.76, p = 0.001). Patients who were not placed in Dolutegravir (DTG) based drug were more likely to interrup treatment (RR: 6.67, 95% CI 3.36–7.04, p < 0.001) compared to those who were taking DTG drug combination. Viral load suppression failure was not significantly found to be a risk factor for interruption in treatment among adolescence in the region (RR: 1.04, 95% CI 0.83–1.30, p = 0.73).

## Discussion

This is a retrospective longitudinal cohort study conducted among HIV-infected adolescents in Tanga, Tanzania. Results showed that the overall interruption in treatment rate was high (26.2%), much higher than required to meet the 95-95-95 targets. Adolescents aged between 15 and 19 years, male sex, those residing from Mheza district, advanced HIV disease, being on ART for long time and those placed in non DTG based drug had significantly higher risk for interruption in treatment. Our study population of HIV-infected adolescents in Tanga was higher enough for whom detailed longitudinal clinical and demographic were available.

The interruption in treatment rate observed in this study population were high and somewhat consistent with other studies carried out in some African countries and even outside the continent. Other estimates of interruption in treatment among HIV-infected adolescents in Sub-Saharan African countries including Cameroon, Kenya, Tanzania and South Africa have been reported ranging from 13.4 to 42.2% [4, 14–16]. However, compared to our study, low propotion of interruption in treatment of 4.3% was reported in Asian regional cohort study incoprporating six countries in Asia, 9.2% among Indian adolescents [17] and 8.4% in Zimbabwe [18]. This wide range of reported proportion of interruption in treatment could be due to differences in the study population and the disparity of follow up time in various studies. Viral load suppression among adolescents during the time of study, fear of discrimination and disclosure is anticipated to cause dropout to treatment and care among our study population. Strengthening the comprehesive tracking system and establishment of adolescents HIV support groups are highly encouraged.

The current study found that the determinants of interruption in treatment were generally similar to those in other African settings. Adolescents aged 15–19 years had a higher risk of interruption in treatment from ART uptake. The finding was inline with previous studies conducted in Ethiopia, South Africa, and sub-Saharan Africa [19–21]. Fear of stigma, peer pressure, growing up independent and discrimination might be a contributor to interruption in treatment among adolescents [8, 22]. In our context, we suspect but cannot confirm without further research that the significantly greater treatment interruption observed among adolescents of the said age group could be caused by their being busy with work or feeling shy about attending the clinics. Clearly, treatment facilities need to focus more on preventing IIT among adolescents to increase their chances of survival and gain them the greatest benefit from treatment and conduct age-specific interventions to reduce interruption in treatment among adolescents in our resource settings.

We also identified increased risk of interruption in treatment among male adolescents in our study which was consistent with some previous studies conducted in Tanzania, Ethiopia and Malawi [23–25]. Nevertheless, a study conducted in 15 ART programs in Africa, Asia, and South America reported no association between gender and interruption in treatment [26]. However, previous findings in Uganda and a study conducted on MTCT-Plus programs in 9 different countries showed male adolescents were protective towards interruption in treatment [27, 28]. The reason for gender gap of interruption in treatment among male adolescents in our study might be attributed to challenges of obidiency, busy with recreational activities, stigma, undocumented transfer to other HIV care clinics and death. This findings advocates for strengthening the linkage to HIV care and counseling, implement adolescent-friendly service approach and necessitates further investigation of cultural and behavioural differences among male adolescents for their better clinical health outcome.

Our study found that sicker patients were more likely to interrupt treatment. Increased risk of interruption in treatment with advanced WHO clinical staging (stage IV) observed in our study was consistent with previous study conducted in Tanzania, India, Kenya and Nzimbabwe [14, 16–18], that adolescents with more advanced disease stage understand the benefit of regular clinic visit, however they can’t stick to patient follow-up schedule may be due to their weak conditions or undocumented transferred out or death. It is important for measures to be put in place that improve retention rates among patients who display advanced symptoms of HIV despite taking ART, since results from populations with high interruption in treatment may be biased. Adolescent patients who are lost during follow-up may be less healthy than the patients who remain in the program and therefore more likely to die, leading to underestimation of death rates. Alternatively, the sickest adolescents on ART may be more highly motivated to sustain regular follow-up while the healthiest, asymptomatic adolescent patients may have extended periods between follow-up visits, given little perceived need. Such patients may be less likely to die and may subsequently return to the follow-up. At a program level, interruption in treatment can make it difficult to evaluate outcomes of treatment and care [12, 29]. High rates of interruption in treatment also diminish treatment options and substantially limit the effectiveness of ART strategies [12]. This findings calls for early test and treat services among adolescents in our resource-constrained settings. However, no association was observed in a study conducted in Kenya between interruption in treatment and advanced WHO stage [16].

This study, like other studies, has found that adolescence placed in second-line ART regimen had reduced risk of of interruption to treatment, our findings agree with studies conducted in Tanzania and Ethiopia [14, 30]. However, our results contradict other studies conducted in Myanmar and Nigeria [31, 32], which reported a likelihood risk of interruption in treatment among patients on a second-line regimen. A study conducted in Nigeria stated an increased risk of interruption in treatment might be caused by adverse effects obtained from second-line drugs [32]. Adolescents in the second-line being protective towards interruption in treatment in our study could be expalined by intensive counseling and close followup given to this group after first line treatment failure by health care givers.

Studies in South Africa and USA show that among adolescents who virally unsupressed the risk of interruption in treatment is high [15, 33]. However, an interesting findings in our study show that viral load suppression failure was not found to be a significant risk factor for interruption in treatment among adolescence in Tanga region. This perhaps is due the fact that adolescents with poor viral suppression has special management package named enhanced adherence counseling where each unsupressed client is paired with the counselor and peer for close followup to support adherence to clinic visits and ART uptake.

Other noteworthy findings were increased risk of interruption in treatment among adolescence who have been placed in non-DTG based regimen of which we have observed limited report of related studies and among adolescents who have been on ART for long time (at least one year from enrollment or ART initiation date). This finding contradict with other studies in African countries. For instance, a study conducted in South Africa show that shorter time on ART (≤ 12 months) was independently associated with higher interruption in treatment [15]. Moreover, prolonged period on ART was protective among adolescents in a retrospective cohort study conducted in Northwest and Southwest of Cameroon [4]. High rate of interruption in treatment in our study might be due attributed to patients who have been on treatment for long period, hence some feel they are healthier or they are get tired of taking drugs or they are in advance disease progression. This calls for a need for healthcare providers to keep track of long term clients on ART in order to reduce likelihood of transmission of HIV in the community.

It is important to note that clinics may tend to invest their limited resources in educating patients who are initiating ART about adherence, rather than in efforts to track patients who have interrupted treatment in their communities and return them to care, further exascerbating the impact of non-retention. Finally, in order to reach the recommended 95-95-95 goals to end the AIDS epidemic by 2030, it is essential that adolescent patients who are initiated with ART remain in care and virally suppressed, the final step of the treatment/prevention cascade [34]. With the goal of all HIV-positives adolescent being on treatment, without adequate retention, there is substantial concern about the emergence of unresolveable drug resistance [35]. It is important from a clinical and programmatic perspective to ensure that HIV care and treatment serive providers are aware of this high risk patient group of adolescents and put in place good and relevant strategies to track and retain them.

Major strengths of this study is the relatively large sample size with inclusion of all the 86 health facilities in the region, providing us substantial power to obtain accurate and reliable estimate over a large number of potential risk factors, adjusted for one another, and the sufficient follow-up time of 24 months. Additionally, the longitudinal nature of this analysis provided an opportunity to assess rate and time of interruption in treatment and associated predictors. The major limitation is that our analysis based on the secondary data from the existing database. It is possible that not all clinical data were correctly captured and that some data were not captured at all, resulting in missing data which however was handled during data analysis but this is a general limitation of using routine data.

## Conclusions

Our study has shown a high incidence of interruption in anti-retroviral treatment among adolescents living with HIV in Tanga region which is still a major concern and likely leading to poor clinical outcomes, and increased drug resistance among ART-initiated adolescents in the region. In addition we found that a high risk of ART treatment interruption is significantly attributed to either among Adolescents aged between 15 and 19 years, a male gender, advanced HIV disease, Muheza district, being on ART for long time and being on a non DTG based drug. Our results highlight the needs for multi-intervention and cross-cutting strategies to address the identified attributing factors among adolescents in care and treatment facilities in the region. This can include designing integrated clinics and youth clubs and camps for adolescents, especially in primary health facilities to increase retention in HIV care services.

## Data Availability

The data used for this study contain sensitive information about the participants. Also, the participants did not provide their approval for the sharing of their information. However, for researchers who meet the requirements for access to confidential data, data are available from the Directorate of National Aids Control Program (contact via nacp@afya.go.tz).
